# Ethno-cultural disparities in mental health during the COVID-19 pandemic: a cross-sectional study on the impact of exposure to the virus and COVID-19-related discrimination and stigma on mental health across ethno-cultural groups in Quebec (Canada)

**DOI:** 10.1192/bjo.2020.146

**Published:** 2020-12-09

**Authors:** Diana Miconi, Zhi Yin Li, Rochelle L. Frounfelker, Tara Santavicca, Jude Mary Cénat, Vivek Venkatesh, Cécile Rousseau

**Affiliations:** Division of Social and Cultural Psychiatry, McGill University, Canada; Department of Epidemiology, Biostatistics, and Occupational Health, McGill University, Canada; Division of Social and Cultural Psychiatry, McGill University, Canada; Department of Epidemiology, Biostatistics, and Occupational Health, McGill University, Canada; School of Psychology (Clinical), University of Ottawa, Canada; UNESCO co-Chair on Prevention of Radicalisation and Violent Extremism, Concordia University, Canada; Division of Social and Cultural Psychiatry, McGill University, Canada

**Keywords:** Pandemic, mental health, sociocultural factors, discrimination and stigma, exposure to virus

## Abstract

**Background:**

Although social and structural inequalities associated with COVID-19 have been documented since the start of the pandemic, few studies have explored the association between pandemic-specific risk factors and the mental health of minority populations.

**Aims:**

We investigated the association of exposure to the virus, COVID-19-related discrimination and stigma with mental health during the COVID-19 pandemic, in a culturally diverse sample of adults in Quebec (Canada).

**Method:**

A total of 3273 residents of the province of Quebec (49% aged 18–39 years, 57% women, 51% belonging to a minority ethno-cultural group) completed an online survey. We used linear and ordinal logistic regression to identify the relationship between COVID-19 experiences and mental health, and the moderating role of ethno-cultural identity.

**Results:**

Mental health varied significantly based on socioeconomic status and ethno-cultural group, with those with lower incomes and Arab participants reporting higher psychological distress. Exposure to the virus, COVID-19-related discrimination, and stigma were associated with poorer mental health. Associations with mental health varied across ethno-cultural groups, with exposed and discriminated Black participants reporting higher mental distress.

**Conclusions:**

Findings indicate sociocultural inequalities in mental health related to COVID-19 in the Canadian context. COVID-19-related risk factors, including exposure, discrimination and stigma, jeopardise mental health. This burden is most noteworthy for the Black community. There is an urgent need for public health authorities and health professionals to advocate against the discrimination of racialised minorities, and ensure that mental health services are accessible and culturally sensitive during and in the aftermath of the pandemic.

The COVID-19 pandemic is affecting social, cultural and economic systems around the world, and mounting evidence suggests profound and concerning negative effects of COVID-19 on mental health, with long-lasting consequences on society.^1–3^ Preliminary reports from the USA and the UK have denounced how individuals that experience structural and social inequities, such as ethnic and racial minorities,^4,5^ are disproportionately exposed to the virus and affected by the pandemic. This is because of systemic social and economic disparities,^6–8^ including poverty, poor housing and inadequate healthcare, and has prompted a call to identify and address sociocultural health disparities in the COVID-19 crisis. Less is known about how such systemic social and economic inequalities, and associated experiences during the pandemic, affect the mental health of vulnerable communities. Indeed, the pandemic has highlighted social, economic and political fractures and injustices within communities and societies, fuelling fear and xenophobic discourses in the general population. As a result, minorities and marginalised groups, who have already been severely affected by the pandemic, have also increasingly become the target of COVID-19-related racialised and discriminatory actions.^5,9–13^ Although conspiracy theories and ‘othering’ processes targeting minorities and at-risk groups are common in pandemics,^12^ empirical evidence on the impact of sociocultural factors and COVID-19-related experiences of exposure, stigma and discrimination on mental health are scarce.

## Discrimination, stigma, exposure and mental health during a pandemic

Discrimination and stigma refer to complex and diverse social processes that exist at the individual, interpersonal and structural levels of society, and represent significant public health concerns.^14^ Stigma refers to the process of unfair treatment of others, and prevents opportunities for equal participation in society for stigmatised groups, fuelling social inequalities.^15^ In the current study, we focus on ‘individual’ stigma, referring to the internalisation of discriminating beliefs and associated feelings of shame, leading to concealment, and on experiences of discrimination as a form of ‘enacted’ stigma.^16^ Prior research documented the overall negative impact of stigma^17–19^ and discrimination^20,21^ on mental health. With regards to exposure, a recent meta-analysis showed that direct exposure to the Ebola virus is linked with more mental distress, although the magnitude of this association may vary according to personal and sociocultural experiences and characteristics.^22^

However, empirical evidence on the relationship of exposure to the virus, pandemic-specific stigma and discrimination with mental health during the COVID-19 pandemic is still limited. The few available empirical studies from the USA indicate that gender, occupation, age, socioeconomic status, being a member of a racial/ethnic minority, being foreign-born and experiencing discrimination are associated with COVID-19-related mental health.^23,24^ Direct exposure to COVID-19 was a risk factor for mental health in a study conducted on the Chinese general population,^25^ and among healthcare workers in China.^2^ Much less is known about the Canadian context or among culturally diverse samples. Given that experiences of exposure to COVID-19 and COVID-19-related stigma and discrimination may play a detrimental role on one's mental health during the present pandemic, empirical studies aimed at shedding light on the contributions of such factors to one's mental health in culturally diverse samples are warranted.

## The Quebec context

In Canada, the first case of COVID-19 was confirmed at the end of January 2020. Although representing just 22.57% of the national population, with >52% of confirmed cases and >64% of deaths, the province of Quebec became the epicentre of the pandemic in Canada.^26^ More than one-third of confirmed cases in Quebec were identified in the city of Montreal, with a disproportionately higher number of individuals diagnosed with COVID-19 residing in diverse, multiracial areas of the city, suggesting cultural and social disparities in rates of COVID-19 infections and deaths.^27^ Specific concerns have been expressed over issues of systemic discrimination and unsafe work conditions, given that healthcare attendants in seniors’ residences and hospitals are mostly racialised (e.g. Black, Asian, Latino and Arab).^27^ Since March 2020, there has been an increase in reported discrimination and xenophobic incidents directed at members of Asian communities in the province, including hate speech, vandalism and physical intimidation on streets and in stores.^27^ It is important to note that Quebec society is demographically and culturally diverse, and 21.9% of its population is foreign-born;^28^ this highlights the importance of investigating social and ethnic disparities during the current health emergency. Information on sociocultural correlates of mental health during the pandemic is critical to inform public health interventions and programmes for at-risk populations at the institutional, community and individual level.

## The current study

This study investigates the association of sociocultural characteristics and pandemic-specific risk factors (i.e. exposure to the virus, COVID-19-related discrimination and stigma) with mental health during the COVID-19 pandemic in a culturally diverse sample of adults in Quebec (Canada). Specifically, we investigate the following: (a) whether sociocultural characteristics (i.e. ethno-cultural group, immigrant generation, income) are associated with mental health; (b) whether exposure to the virus and COVID-19-related discrimination and stigma are associated with mental health, when controlling for relevant sociodemographic variables, including prior mental health and discrimination not related to the pandemic; and (c) whether the association between risk factors and mental health varies across ethno-cultural groups. Based on the limited evidence on sociocultural vulnerabilities during the COVID-19 pandemic, we expected participants with lower economic resources, an immigrant background and/or those who are members of a racialised minority to be at higher risk of mental distress. We expected that exposure to the virus and experiencing COVID-19-related discrimination and stigma would be negatively associated with mental health, and that the magnitude of these relationships would be stronger among those racialised minority groups most affected by the pandemic.

## Method

### Participants and procedure

A total of 3273 residents of the province of Quebec, aged ≥18 years, completed an online survey (see Table 1). Participants were randomly selected from the Leo panel (Léger Opinion), which includes >400 000 Canadian households. To get to a culturally diverse sample, respondents who matched the ‘visible minority’ profile were targeted in the panel based on the ethnic profiling information available in the Leo panel. The research project was presented as a study about COVID-19 and social distancing. Participants completed the survey in either French or English, between 1 June 2020 and 23 June 2020. Participation was voluntary and confidential. All participants received from 50 cents to $2 in compensation, depending upon length of time taken to complete the survey (average completion time of 12 min), and provided electronic informed consent. A total of 8825 invitation emails were sent. The response rate was 37%. The authors assert that all procedures contributing to this work comply with the ethical standards of the relevant national and institutional committees on human experimentation and with the Helsinki Declaration of 1975, as revised in 2008. All procedures involving human participants were approved by the McGill Faculty of Medicine Institutional Review Board (Approval no. A05-B25-20A 20-05-005) .

**Table 1:** Sociocultural characteristics of participants and descriptive statistics of outcomes across sociocultural variables

	Total sample		HSCL-10			Impact of COVID-19 on mental health				
	*n*	%	*n*	Mean (s.d.)	*P*-value	*n*	Prevalence			*P*- value
							‘A great deal’	‘A little bit’	‘None’	
Age	3273		3195		<0.001	3252				<0.001
18–39 years	1611	49.22	1555	1.85 (0.64)		1594	19.26%	55.21%	25.53%	
40–59 years	994	30.37	977	1.64 (0.56)		991	12.41%	49.65%	37.94%	
≥60 years	668	20.41	663	1.47 (0.45)		667	6.15%	40.78%	53.07%	
Gender	3273		3195		<0.001	3252				<0.001
Male	1418	43.32	1386	1.61 (0.58)		1410	11.06%	44.89%	44.04%	
Female	1855	56.68	1809	1.78 (0.60)		1842	17.10%	54.89%	28.01%	
Race/ethnicity	3273		3195		<0.001	3252				<0.001
White	1606	49.07	1583	1.63 (0.56)		1599	12.01%	48.72%	39.27%	
East Asian	249	7.61	243	1.70 (0.59)		246	10.98%	57.32%	31.71%	
South Asian	96	2.93	90	1.81 (0.64)		93	21.51%	52.69%	25.81%	
Black	692	21.14	669	1.75 (0.63)		687	18.05%	47.74%	34.21%	
South-East Asian	119	3.64	115	1.78 (0.67)		119	15.13%	59.66%	25.21%	
Arab	450	13.75	434	1.86 (0.61)		447	17.67%	53.02%	29.31%	
Other	61	1.86	61	1.93 (0.72)		61	18.03%	63.93%	18.03%	
Religion	3176		3106		<0.001	3156				.003
Christianism	1626	51.20	1594	1.67 (0.58)		1616	13.37%	48.89%	37.75%	
Islam	378	11.90	362	1.83 (0.59)		372	16.40%	52.69%	30.91%	
Judaism	135	4.25	134	1.61 (0.59)		134	11.19%	48.51%	40.30%	
Atheism	906	28.53	890	1.73 (0.61)		904	15.15%	52.77%	32.08%	
Other	131	4.12	126	1.80 (0.70)		130	18.46%	51.54%	30.00%	
Main language	3273		3195		<0.001	3252				<0.001
French	1984	60.62	1941	1.67 (0.57)		1973	13.38%	48.91%	37.71%	
English	534	16.32	518	1.77 (0.65)		530	15.66%	52.64%	31.70%	
Both	755	23.07	736	1.77 (0.63)		749	16.56%	53.40%	30.04%	
Immigrant generation	3221		3155		<0.001	3208				<0.001
First	1167	36.23	1137	1.72 (0.59)		1163	14.36%	50.64%	35.00%	
Second	668	20.74	655	1.82 (0.65)		665	17.14%	56.54%	26.32%	
Third or more	1386	43.03	1363	1.64 (0.57)		1380	13.19%	47.75%	39.06%	
Education	3229		3163		0.101	3215				0.487
High school or less	476	14.74	463	1.76 (0.61)		473	14.16%	51.59%	34.25%	
Technical degree/some college or university	1218	37.72	1189	1.71 (0.62)		1212	15.59%	50.17%	34.24%	
University degree or above	1535	47.54	1511	1.69 (0.58)		1530	13.73%	50.65%	35.62%	
Household income	2928		2877		<0.001	2918				0.002
≤$19 999	293	10.01	277	1.90 (0.67)		285	18.60%	50.88%	30.53%	
$20 000–$39 999	447	15.27	437	1.81 (0.65)		446	18.16%	48.65%	33.18%	
$40 000–$59 999	604	20.63	596	1.75 (0.59)		603	15.92%	52.57%	31.51%	
$60 000–$79 999	492	16.80	483	1.69 (0.61)		492	12.80%	47.97%	39.23%	
$80 000–$99 999	382	13.05	380	1.65 (0.56)		382	13.09%	50.26%	36.65%	
≥$100 000	710	24.25	704	1.60 (0.55)		710	11.41%	51.27%	37.32%	
Employment	3219		3154		0.830	3206				<0.001
Employed, essential worker	1046	32.49	1023	1.71 (0.60)		1043	16.20%	52.64%	31.16%	
Employed, non-essential worker	886	27.52	873	1.71 (0.59)		884	14.14%	52.38%	33.48%	
Unemployed	1287	39.98	1258	1.70 (0.60)		1279	13.45%	47.46%	39.09%	
Household size	3193		3127		<0.001	3178				0.022
One person	605	18.95	590	1.69 (0.60)		600	15.67%	49.00%	35.33%	
Two people	1077	33.73	1061	1.63 (0.54)		1073	12.30%	49.58%	38.12%	
Three people	594	18.60	580	1.71 (0.59)		593	14.00%	52.28%	33.73%	
Four people	573	17.95	561	1.80 (0.64)		569	15.82%	52.90%	31.28%	
Five or more people	344	10.77	335	1.78 (0.64)		343	18.08%	48.10%	33.82%	
Geographical location	3180		3115		<0.001	3166				<0.001
Greater Montreal region	2176	68.43	2126	1.73 (0.61)		2166	15.28%	51.62%	33.10%	
Outside Greater Montreal region	1004	31.57	989	1.64 (0.55)		1000	12.00%	47.50%	40.50%	
Non-COVID-19-related discrimination	3199		3136		<0.001	3185				<0.001
Yes	838	26.20	808	1.98 (0.67)		832	22.60%	51.44%	25.96%	
No	2361	73.80	2328	1.61 (0.54)		2353	11.27%	50.38%	38.35%	
COVID-19 exposure	3231		3163		<0.001	3214				<0.001
Yes	920	28.47	908	1.83 (0.65)		917	19.30%	52.78%	27.92%	
No	2311	71.53	2255	1.66 (0.57)		2297	12.45%	49.54%	38.01%	
Mental health before COVID-19	3243		3182		<0.001	3237				<0.001
Excellent	1865	57.51	1840	1.49 (0.49)		1865	9.17%	41.29%	49.54%	
Average	1120	34.54	1093	1.92 (0.57)		1117	17.73%	66.61%	15.67%	
Poor	258	7.96	249	2.37 (0.64)		255	38.82%	47.84%	13.33%	
COVID-19-related stigma (median)	3217		3157		<0.001	3208				<0.001
≤4	1664	51.73	1642	1.62 (0.57)		1663	13.23%	47.20%	39.57%	
>4	1553	48.27	1515	1.81 (0.62)		1545	15.60%	54.56%	29.84%	
COVID-19-related discrimination	3184		3124		<0.001	3169				<0.001
Yes	551	17.31	529	2.01 (0.68)		546	21.98%	53.48%	24.54%	
No	2633	82.69	2595	1.64 (0.56)		2623	12.81%	49.87%	37.32%	
Total	3273		3195	1.71 (0.60)		3252	14.48%	50.55%	34.96%	

The ‘Other’ ethno-cultural cohort grouped participants who self-identified as West Asian (*n* = 30), Latin American (*n* = 27) and who responded ‘other’ to the question on their ethno-cultural group (*n* = 4). The *P*-value of the univariate effect of each sociocultural variable and predictor on outcomes is reported (*n* = 3273). HSCL-10, Hopkins Symptom Checklist-10.

### Measures

#### Mental health

Mental health was assessed with the Hopkins Symptom Checklist-10 (HSCL-10),^29^ comprising six items measuring symptoms of depression and four items measuring symptoms of anxiety. Participants are asked to rate on a Likert scale from 1 (not at all) to 4 (extremely), how much they were bothered by the reported symptoms during the past week. Symptom severity is computed by averaging responses on the items (range 1–4), with higher scores indicating higher distress. Cronbach's *α* and McDonald's *ω* were both 0.89 in our sample.

Perceived impact of COVID-19 on mental health is a categorical variable (none, a little bit, a great deal), measured by participant responses to the question, ‘How much has the COVID-19 epidemic affected your mental health?’.

Prior exposure to COVID-19 was measured via five questions (yes/no response format), to investigate whether the participant had been diagnosed with COVID-19 and if they knew anyone around them, among their neighbours, friends and/or within their household/family, who had been diagnosed with COVID-19 in the past month. Responses were categorised into a binary variable (yes/no), with participants who replied yes to at least one of the questions considered as having been exposed to COVID-19.

#### COVID-19-related discrimination

All participants were asked to report experiences of perceived discrimination (if any) in the past month as a result of their presumed COVID-19 status, based on a questionnaire developed by Williams et al^30^ and adapted to the present health emergency context. Responses were categorised into a binary variable (yes/no).

#### COVID-19-related stigma

Participants indicated on a seven-point Likert scale how much they agreed with the following statements: If a member of my family became ill with COVID-19, I would want it to remain secret; If I became ill with COVID-19, I would want it to remain secret. Responses to the two questions were summed, with higher scores indicating greater stigma (range 2–14).

#### Sociocultural variables

Participants provided information on their age (18–39, 40–59 or ≥60 years), gender (male, female or other), education (high school or less, technical degree or some college/university, university degree and above), household income (≤$19 999, $20 000–$39 999, $40 000–$59 999, $60 000–$79 999, $80 000–$99 999 or ≥$100 000), number of people in the household (one, two, three, four or five or more), immigrant generation (first-, second- or third-generation immigrant and above), religion (Christianism, Islam, Judaism, Atheism or other), race/ethnicity (White, East Asian, South Asian, Black, South-East Asian, Arab or other), language (French, English or both), employment (unemployed, employed and designated as an essential worker by the Quebec government, or employed but not designated as an essential worker). Perceived discrimination not related to COVID-19 in the past month was measured as a binary variable (yes/no). Self-reported mental health before the pandemic was assessed with one item, on a three-point Likert scale (poor, average or excellent).

### Data analysis

Descriptive information for the sample was summarised with counts and proportions for categorical variables, and means and s.d. for continuous variables, as well as univariate analysis to examine differences in mental health according to sociocultural variables. Missing values for both continuous and categorical variables were imputed with multiple imputations by chained equations (*n* = 10).^31^ Sensitivity analysis suggested that missing data and multiple imputations did not alter the observed patterns of associations. As the total HSCL-10 scale was not normally distributed, we extracted factor scores of the HSCL-10 latent function via a confirmatory factor analysis on the HSCL-10 items, testing a single latent variable model, using a diagonally weighted least squares method. Factor scores had a univariate distribution closer to normal than raw scores, and were therefore included as the outcome of interest in the subsequent multivariate models. Total stigma scores were standardised to a mean of 0 and an s.d. of 1, to facilitate interpretation, allowing for inference of the effect of a 1-s.d. increase in stigma on HSCL-10 scores. Regression analyses were conducted in three steps: first, we tested linear and ordinal logistic regression models to assess the relationship between sociocultural variables and mental health; next, we tested linear and ordinal logistic regression models, controlling for the relevant sociodemographic variables, to assess the impact of prior exposure to COVID-19 and COVID-19-related discrimination and stigma on mental health; and finally, in the same models, we included a two-way interaction between each predictor (i.e. exposure, COVID-19-related discrimination and stigma) and race/ethnicity, to explore potential effect modification by ethno-cultural group. The threshold for statistical significance was set to 0.05 (two-sided tests). R software version 4.0.3 for Apple (R Foundation for Statistical Computing, Vienna, Austria; see https://www.R-project.org/) was used in all analyses.^32^

## Results

Descriptive statistics of the sample across sociocultural variables at the univariate level are reported in Tables 1 and 2. In terms of mental health, all sociocultural variables except education and employment were significantly associated with HSCL-10 scores. All variables except education were significantly associated with self-reported impact of COVID-19 on mental health at the univariate level (Table 1). Participants aged 18–39 years, first- and second-generation immigrants, essential workers, people living in Montreal and in households of three or more people, and participants who experienced discrimination not related to COVID-19 reported higher prevalence of exposure to the virus and COVID-19-related discrimination, and higher endorsement of COVID-19-related stigma. Black, Arab and South Asian participants had a higher prevalence of exposure, whereas Asian and Black participants reported more COVID-19-related discrimination and stigma. Muslim participants were the religious group most exposed to the virus, followed by Christian participants. Muslim participants and participants who identified with ‘other’ in terms of religion reported higher COVID-19-related discrimination. Anglophone participants were less exposed to the virus, but Francophone participants reported less discrimination because of COVID-19. Participants with an income >$40 000 were more exposed to the virus, whereas participants with an income <$20 000 reported higher stigma and more COVID-19-related discrimination. Participants who self-reported poor mental health before the pandemic also reported higher stigma; participants who were exposed to the virus reported higher stigma and higher prevalence of COVID-19-related discrimination. Participants who reported higher stigma (above median) also reported a higher prevalence of exposure and COVID-19-related discrimination. Neither education nor gender were associated with exposure, stigma or COVID-19-related discrimination (see Table 2).

**Table 2:** Descriptive statistics of study variables across sociocultural variables

	Exposure to COVID-19			COVID-19-related stigma			COVID-19-related discrimination		
	*n*	Prevalence	*P*-value	*n*	Mean (s.d.)	*P*-value	*n*	Prevalence	*P*-value
Age	3231		<0.001	3217		<0.001	3184		<0.001
18–39 years	1589	34.11%		1576	6.45 (3.97)		1551	24.37%	
40–59 years	978	25.66%		982	5.29 (3.65)		974	10.88%	
≥60 years	664	19.13%		659	4.16 (3.15)		659	10.17%	
Gender	3231		0.059	3217		0.533	3184		0.924
Male	1401	26.77%		1392	5.68 (3.81)		1381	17.38%	
Female	1830	29.78%		1825	5.59 (3.83)		1803	17.25%	
Race/ethnicity	3231		<0.001	3217		<0.001	3184		<0.001
White	1597	23.79%		1586	4.77 (3.40)		1575	10.03%	
East Asian	247	21.86%		244	6.54 (3.79)		245	31.43%	
South Asian	94	28.72%		89	6.29 (4.00)		93	30.11%	
Black	674	38.72%		678	6.60 (4.11)		666	24.32%	
South-East Asian	116	30.17%		118	6.24 (4.14)		117	25.64%	
Arab	444	33.56%		444	6.41 (4.01)		427	19.20%	
Other	59	23.73%		58	5.50 (3.90)		61	22.95%	
Religion	3137		0.024	3125		<0.001	3097		<0.001
Christianism	1598	29.79%		1606	5.43 (3.78)		1595	16.30%	
Islam	372	33.87%		368	6.99 (4.05)		353	24.36%	
Judaism	135	24.44%		132	4.78 (3.45)		133	5.26%	
Atheism	902	25.72%		891	5.31 (3.60)		886	14.67%	
Other	130	26.15%		128	6.59 (4.27)		130	39.23%	
Main language	3231		<0.001	3217		0.065	3184		<0.001
French	1958	29.01%		1953	5.50 (3.82)		1921	14.37%	
English	527	22.20%		520	5.87 (3.87)		524	22.71%	
Both	746	31.50%		744	5.79 (3.78)		739	21.11%	
Immigrant generation	3182		<0.001	3173		<0.001	3142		<0.001
First	1147	28.51%		1145	6.07 (3.99)		1132	20.58%	
Second	660	38.18%		661	6.34 (3.93)		651	23.35%	
Third or more	1375	24.36%		1367	4.88 (3.47)		1359	11.41%	
Education	3190		0.943	3184		0.231	3151		0.117
High school or less	468	28.85%		469	5.89 (3.97)		462	19.91%	
Technical degree/some college or university	1201	28.81%		1191	5.55 (3.75)		1182	17.94%	
University degree or above	1521	28.27%		1524	5.57 (3.82)		1507	15.99%	
Household income	2895		0.012	2889		<0.001	2859		<0.001
≤$19 999	290	23.10%		282	6.41 (4.00)		278	27.34%	
$20 000–$39 999	438	24.43%		440	5.74 (3.98)		439	23.46%	
$40 000–$59 999	594	28.79%		595	5.61 (3.72)		585	18.63%	
$60 000–$79 999	489	30.67%		487	5.88 (3.94)		482	16.80%	
$80 000–$99 999	378	33.86%		380	5.61 (3.84)		374	12.57%	
≥$100 000	706	29.46%		705	5.12 (3.59)		701	12.98%	
Employment	3181		<0.001	3172		<0.001	3140		<0.001
Employed, essential worker	1031	37.73%		1038	6.07 (4.01)		1027	22.30%	
Employed, non-essential worker	878	24.26%		869	5.51 (3.63)		864	13.08%	
Unemployed	1272	24.06%		1265	5.26 (3.73)		1249	16.01%	
Household size	3154		<0.001	3145		<0.001	3118		0.013
One person	596	21.31%		586	5.38 (3.70)		587	15.16%	
Two people	1070	25.61%		1063	5.20 (3.69)		1058	14.56%	
Three people	584	33.90%		588	5.91 (4.00)		582	19.24%	
Four people	563	29.66%		565	5.80 (3.83)		557	18.13%	
Five or more people	341	38.12%		343	6.14 (3.83)		334	21.26%	
Geographical location	3144		<0.001	3133		0.002	3102		0.002
Greater Montreal region	2150	32.88%		2142	5.71 (3.87)		2126	18.34%	
Outside Greater Montreal region	994	18.71%		991	5.26 (3.63)		976	13.93%	
Non-COVID-19-related discrimination	3163		<0.001	3155		<0.001	3151		<0.001
Yes	819	40.78%		824	6.70 (4.06)		816	50.86%	
No	2344	24.53%		2331	5.21 (3.65)		2335	5.52%	
COVID-19 exposure				3181		<0.001	3145		<0.001
Yes				914	6.00 (4.00)		900	27.33%	
No				2267	5.46 (3.73)		2245	12.92%	
Mental health before COVID-19	3207		0.131	3200		<0.001	3162		<0.001
Excellent	1846	27.63%		1841	5.31 (3.81)		1820	13.85%	
Average	1106	28.84%		1106	5.89 (3.75)		1092	19.41%	
Poor	255	33.73%		253	6.58 (3.96)		250	31.60%	
COVID-19-related stigma (median)	3181		0.002				3141		<0.001
≤4	1649	26.38%					1645	11.19%	
>4	1532	31.27%					1496	23.80%	
COVID-19-related discrimination	3145		<0.001	3141		<0.001			
Yes	536	45.90%		540	7.29 (4.11)				
No	2609	25.07%		2601	5.22 (3.65)				
Total	3231	28.47%		3217	5.63 (3.82)		3184	17.31%	

*P*-value of the univariate effect of each sociocultural variable on predictors is reported (*n* = 3273).

In multivariate models, women and participants aged between 18 and 39 years reported worse mental health across both outcomes. Arab participants had higher HSCL-10 scores and reported a greater impact of the pandemic on their mental health than other racial/cultural groups. East Asian participants reported lower HSCL-10 scores compared with other ethno-cultural groups. Participants who reported poorer mental health before COVID-19 scored higher on the HSCL-10 scale and reported a stronger impact of the pandemic on mental health. Non-COVID-19-related discrimination was also associated with both mental health outcomes. Individuals with a lower household income (<$100,000), and those living with three people in the same household, had higher HSCL-10 scores, but not more perceived impact of COVID-19 on mental health, than those living alone. Participants living in the Greater Montreal area reported greater impact of the pandemic on their mental health than those living in other parts of Quebec. Employment, education, generation, language and religion were not associated with either mental health outcome at the multivariate level (see Table 3). Differences in the associations of sociocultural variables with mental health outcomes at the univariate and multivariate levels may be partially explained by issues of collinearity among variables (see Supplementary material available at https://doi.org/10.1192/bjo.2020.146).

**Table 3:** Results of multivariate linear and ordered logistic regression models on HSCL-10 total scores and impact of COVID-19 on mental health (n = 3273)

Variables	HSCL-10 total (factor scores)				Impact of COVID-19 on mental health		
	*B*	95% CI	Omnibus *F* (d.f.)	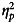	Proportional odds ratio	95% CI	Likelihood ratio *χ*^2^ (d.f.)
Gender			37.896 (1, 17 245.327)***	0.012			44.477 (1, 41 745.532)***
Male	Reference				1		
Female	0.172***	0.117–0.227			1.621***	1.404–1.871	
Age, years			23.513 (2, 2172.733)***	0.015			45.230 (2, 13 7981.981)***
18–39	Reference				1		
40–59	−0.150***	−0.217 to −0.084			0.745**	0.630–0.883	
≥60	−0.307***	−0.400 to −0.214			0.451***	0.356–0.573	
Race/ethnicity			5.298 (6, 1191.333)**				13.598 (6, 2667.706)*
White	Reference				1		
East Asian	−0.164*	−0.292 to −0.036			0.785	0.564–1.094	
South Asian	0.004	−0.194 to 0.201			1.372	0.822–2.289	
Black	−0.080	−0.183 to 0.024			0.965	0.739–1.261	
South-East Asian	−0.044	−0.214 to 0.126			1.097	0.712–1.689	
Arab	0.191**	0.069–0.313			1.391*	1.014–1.908	
Other	0.093	−0.114 to 0.299			1.370	0.808–2.323	
Religion			0.236 (4, 1725.438)	<0.001			2.071 (4, 1519.545)
Christianism	Reference				1		
Islam	−0.042	−0.154 to 0.070			0.833	0.624–1.112	
Judaism	−0.046	−0.198 to 0.105			0.962	0.649–1.426	
Atheism	−0.019	−0.085 to 0.047			1.010	0.850–1.200	
Other	−0.035	−0.196 to 0.125			0.928	0.607–1.419	
Main language			1.746 (2, 8159.802)	0.001			0.974 (2, 22 662.688)
French	Reference				1		
English	0.074	−0.012 to 0.160			1.115	0.893–1.391	
Both	−0.007	−0.076 to 0.062			1.024	0.855–1.227	
Immigrant generation			0.392 (2, 1985.614)	<0.001			2.126 (2, 9562.608)
First	Reference				1		
Second	0.029	−0.049 to 0.108			1.096	0.899–1.336	
Third or more	0.038	−0.057 to 0.133			1.188	0.926–1.523	
Education			0.737 ( 2, 3631.138)	<0.001			0.705 (2, 7634.225)
High school or less	Reference				1		
Technical degree/some college or university	−0.023	−0.108 to 0.062			1.098	0.885–1.362	
University degree or above	0.016	−0.070 to 0.102			1.052	0.845–1.309	
Household income			6.256 (5, 753.312)***	0.011			8.164 (5, 3334.008)
≤$19 999	Reference				1		
$20 000–$39 999	0.059	−0.062 to 0.179			1.107	0.815–1.503	
$40 000–$59 999	−0.023	−0.142 to 0.096			1.186	0.886–1.588	
$60 000–$79 999	−0.062	−0.185 to 0.060			0.887	0.642–1.225	
$80 000–$99 999	−0.127	−0.265 to −0.012			0.946	0.682–1.312	
≥$100 000	−0.193**	−0.319 to −0.068			0.971	0.711–1.324	
Household size			2.588 (4, 3644.241)*	0.003			2.562 (4, 13 385.440)
One person	Reference				1		
Two people	0.003	−0.079 to 0.085			0.969	0.784–1.197	
Three people	0.011	−0.082 to 0.104			0.840	0.661–1.069	
Four people	0.119*	0.020 to 0.219			0.948	0.741–1.214	
Five or more people	0.054	−0.058 to 0.166			0.899	0.677–1.194	
Employment			1.809 (2, 1948.613)	0.001			1.439 (2, 93 757.358)
Unemployed	Reference				1		
Employed, essential worker	−0.062	−0.135 to −0.011			1.117	0.929–1.342	
Employed, non-essential worker	−0.006	−0.083 to 0.070			1.076	0.887–1.305	
Geographical location			1.616 (1, 11 208.481)	<0.001			5.508 (1, 15 377.989)*
Outside Greater Montreal region	Reference				1		
Greater Montreal region	0.041	−0.022 to 0.104			1.214*	1.030–1.431	
Discrimination not related to COVID-19			120.889 (1, 2806.166)***	0.038			25.718 (1, 12 797.430)***
No	Reference				1		
Yes	0.362***	0.297–0.426			1.530***	1.295–1.807	
Mental health before COVID-19			307.119 (2, 20 170.448)***	0.162			284.748 (2, 8563.222)***
Excellent	Reference				1		
Average	0.571***	0.511–0.631			3.056***	2.611–3.575	
Poor	1.065***	0.961 to 1.168			5.895***	4.465–7.784	

HSCL-10, Hopkins Symptom Checklist-10.

*P < 0.05, **P < 0.01, ***P < 0.001.

Prior exposure to the virus was associated with HSCL-10 scores and self-reported impact of COVID-19 on mental health. Both COVID-19-related discrimination and stigma were associated with higher scores on the HSCL-10. Neither COVID-19-related discrimination nor reported stigma were associated with perceived impact of COVID-19 on mental health (Table 4). The magnitude of the relationship between exposure to the virus, experiencing COVID-19-related discrimination and HSCL-10 scores was strongest among participants who self-identified as Black and White. Although the interaction effect between COVID-19-related stigma and HSCL-10 scores was not statistically significant, higher perceived stigma was associated with worse mental health among South Asian and Black participants. The effect of exposure to the virus and COVID-19-related discrimination and stigma on the impact of COVID-19 on mental health did not vary across ethno-cultural groups (all *P* > 0.05). However, participants who self-identified as White and Black reported a greater impact of COVID-19 on their mental health when exposed to the virus, compared with those not exposed (see Table 5).

**Table 4:** Effects of exposure to COVID-19 and COVID-19-related discrimination and stigma on HSCL-10 total scores, and impact of COVID-19 on mental health in multivariate linear and ordered logistic regression models (*n* = 3273)

Variables	HSCL-10 total (factor scores)				Impact of COVID-19 on mental health		
	*B*	95% CI	Omnibus *F* (d.f.)	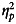	Proportional odds ratio	95% CI	Likelihood ratio *χ*^2^ (d.f.)
Exposure to COVID-19			15.12 (1, 1705.007)***	0.005			15.063 (1, 9398.739)***
No	Reference				1		
Yes	0.122***	0.061–0.183			1.360***	1.161–1.593	
COVID-19-related discrimination			12.395 (1, 1129.500)***	0.004			0.850 (1, 1480.883)
No	Reference				1		
Yes	0.155***	0.068–0.241			1.102	0.880–1.380	
COVID-19-related stigma	0.064***	0.036–0.092	20.541 (1, 9510.511)***	0.007	1.058	0.984–1.138	2.389 (1, 31 145.131)

Both models included sociodemographic variables significant at the *P* < 0.05 level in Table 3 as covariates. HSCL-10, Hopkins Symptom Checklist-10.

*P < 0.05, **P < 0.01, ***P < 0.001.

**Table 5:** Results from moderation (interaction) analyses: associations of exposure to COVID-19 and COVID-19-related discrimination and stigma with total HSCL-10 scores and impact of COVID-19 on mental health, stratified by ethnocultural group (*n* = 3273)

		Predictor					
HSCL-10		Exposure to COVID-19		COVID-19-related discrimination		COVID-19-related stigma	
Moderator		Estimate	95% CI	Estimate	95% CI	Estimate	95% CI
Ethno-cultural group	White	0.149***	0.062–0.237	0.167*	0.032–0.303	0.036	−0.006 to 0.079
	East Asian	0.157	−0.071 to 0.385	−0.520	−0.262 to 0.158	0.036	−0.059 to 0.132
	South Asian	−0.171	−0.519 to 0.177	0.125	−0.221 to 0.471	0.181*	0.026–0.335
	Black	0.246***	0.126–0.366	0.324***	0.182–0.466	0.111***	0.057–0.165
	South-East Asian	−0.134	−0.449 to 0.180	0.102	−0.226 to 0.429	0.113	−0.018 to 0.245
	Arab	−0.029	−0.183 to 0.124	0.047	−0.150 to 0.245	0.048	−0.020 to 0.117
	Other	−0.143	−0.604 to 0.318	−0.099	−0.556 to 0.358	−0.054	−0.245 to 0.1373
	*P-*interaction	0.019		0.050		0.181	
Impact of COVID-19 on mental health							
Moderator		Proportional odds ratio	95% CI	Proportional odds ratio	95% CI	Proportional odds ratio	95% CI
Ethno-cultural group	White	1.328*	1.054–1.673	0.860	−0.599 to 1.233	1.045	0.932–1.171
	East Asian	1.159	0.649–2.070	1.120	0.661–1.899	0.970	0.760–1.239
	South Asian	1.375	0.567–3.331	2.127	0.850–5.321	1.311	0.883–1.945
	Black	1.783***	1.303–2.440	1.378	0.949–1.999	1.122	0.974–1.293
	South-East Asian	0.657	0.303–1.422	0.805	0.345–1.881	1.147	0.824–1.596
	Arab	1.234	0.833–1.829	1.236	0.750–2.038	1.006	0.842–1.202
	Other	1.265	0.386–4.146	0.682	0.220–2.113	0.829	0.511–1.345
	*P-*interaction	0.317		0.311		0.700	

Separate models for each mental health outcome were implemented. Each interaction was tested in separate models. All models presented with HSCL-10 as outcome included age, gender, income, household size, non-COVID-19-related discrimination and prior mental health as covariates. All models presented with impact of COVID-19 on mental health as outcome included age, gender, non-COVID-19-related discrimination, geographical location and prior mental health as covariates. HSCL-10, Hopkins Symptom Checklist-10.

*P < 0.05, **P < 0.01, ***P < 0.001.

## Discussion

Our study sheds light on sociocultural correlates of mental health during the COVID-19 pandemic and highlights the contribution of exposure to the virus and COVID-19-related discrimination and stigma on mental health in a culturally diverse sample of adults. In addition, the association of the hypothesized risk factors with mental health varied across ethno-cultural groups.

As expected, socioeconomic status (in terms of income and household size) and race/ethnicity were both associated with mental health, beyond the contributions of prior mental health, experiences of discrimination not related to COVID-19 and other sociodemographic variables. Participants living in a household with a greater number of people reported higher mental distress, as did participants who declared a lower income. This suggests that socioeconomic hardship represents a risk factor for one's mental health during the present pandemic. Participants who belonged to the Arab ethno-cultural group reported the worst mental health outcomes, whereas participants who self-identified as East Asian reported the best mental health across sociocultural groups. These findings mirror results from the Quebec Cultural Communities Survey.^33^ Such results may be attributed to a combination of both variations in cultural norms around reporting mental health issues (i.e. East Asian participants may be less likely to express distress than other cultural groups), as well as actual differences in mental health across ethno-cultural groups, and are consistent with the literature before the pandemic. Of interest, women and younger participants reported worse mental health, suggesting that these groups may be suffering more from the negative consequences of the pandemic. The fact that immigrant status in terms of first-, second- or third-generation immigrant was not associated with mental health in our study at the multivariate level suggests that identifying as part of a minority group may be more important to mental health than migration status. Possible explanations for this include ‘the immigrant paradox’, whereby first-generation immigrants have fewer mental health problems compared with their native-born offspring,^34^ and the ‘healthy immigrant effect’, in which recent immigrants have good mental health relative to the host population despite higher levels of exposure to adversity.^35^ However, our sample did not include many asylum seekers, refugees and recent immigrants with a lower education level, reported to be at increased risk during the pandemic.^36^

Exposure to COVID-19, experiencing COVID-19-related discrimination and reporting higher levels of COVID-19-related stigma contributed to higher mental distress. Of interest, 17.3% of the sample reported having experienced COVID-19-related discrimination, with the highest prevalence reported by East and South Asian participants. This is not surprising in light of the observed anti-Chinese rhetoric online, and the rapid increase in the number of reports of in-person racist acts against Asian participants in North America.^10^ In terms of exposure to the virus, Black (38.72%), Arab (33.56%) and South Asian (28.72%) communities were among the most exposed ethno-cultural groups, mirroring the composition of the essential workforce in the province, with Black, Asian, Latino and Arab residents overrepresented in the health sector as healthcare attendants in seniors’ residences and hospitals.^27^ Such results provide preliminary evidence in the Canadian context that aligns with reports from the UK and USA, which indicate that communities of colour are disproportionately affected by COVID-19 because of social and economic disparities, including poverty, poor housing and inadequate healthcare.^13^

The association of both exposure to COVID-19 and having experienced at least one episode of COVID-19-related discrimination with mental health varied across ethno-cultural groups. Of importance, Black participants reported the worst mental health outcomes when exposed to the virus and/or to COVID-19-related discrimination, compared with other sociocultural groups. In other words, one's mental health depended on experiences of exposure/discrimination: both exposure and discrimination had a differential effect among ethno-cultural groups, putting Black participants at higher risk of mental distress. Such results suggest that sociocultural inequalities during the pandemic are relevant to mental health outcomes, as well as other health disparities.^5^ In light of the high rates of COVID-19-related hospital admission and mortality among Black Americans in the USA,^37^ and despite the absence of Canadian statistics on ethno-racial rates of morbidity and mortality, these results are not surprising, and align with lessons learned from previous pandemics^38,39^ and well-established documentation of the mental health needs of Black Americans.^40,41^ They clearly indicate that race-conscious and culturally competent interventions, which consider factors such as discrimination and historical and racial trauma, are urgently needed.^42^ Obstacles to access public health and social services as well as protective factors, including community- and culture-specific coping strategies, also need to be considered when planning a concerted response in a time of pandemic. The need for multi-stakeholder interventions that use socio-pedagogical approaches to counter discrimination, through development of prosocial behaviours and moral engagement,^43^ should also be considered as complementary to those adopted by mental health practitioners. Sustained multi-sectoral work in the fields of social services, public health and education that magnifies marginalised communities lived experiences of discrimination is essential in creating dialogic platforms that encourage perspective-taking, and build empathy as cogent outcomes of citizen education initiatives.^44,45^ In addition, to sustainably empower marginalised communities and help build resilience against discrimination, specific attention must be paid to the intersections of identities – gender, sexual, racial and otherwise – thereby highlighting the differential effects of prejudicial acts.

Exposure to the virus was significantly associated with worse mental health outcomes among White participants at a statistical level (*P* < 0.05). Likewise, COVID-19-related discrimination was associated with higher HSCL-10 scores also among White participants. These findings indicate that White participants’ mental health was significantly affected by COVID-19-related experiences such as exposure and discrimination. This is not surprising: although studies rarely scrutinise it in majority groups, discrimination is a heterogeneous phenomenon stemming from individual and group differences, and is always hurtful. Members of the majority group may take their privileges for granted and, because of that, may be on average more likely to expect protection and justice from their environment, and less prepared to endure discrimination. However, at a methodological level, it is important to consider that these statistically significant effects may be attributable to the large sample size of the White ethno-cultural group in our study. This hypothesis is supported by the fact that regression coefficients of the association between exposure/discrimination and mental health among White participants are very similar to those reported across other smaller ethno-cultural groups (which did not, however, reach the 0.05 statistical threshold used in the present study), with the exception of the estimates for Black participants. Overall, these findings, with a closer look at estimates beyond *P*-values, underline that exposure to COVID-19 and related discrimination are risk factors that should not be underestimated across any ethno-cultural group during the present pandemic, although the Black community seems to be at increased risk of mental distress in the present health emergency. Future studies are warranted to shed more light on these issues.

Some differences emerged in terms of findings for each mental health outcome. This indicates that the self-reported impact of the pandemic on mental health and the HSCL-10 scale measure different constructs that are associated, but not overlapping. Specifically, our findings suggest that subjective single-item measures of the impact of COVID-19 on mental health are more independent to COVID-19-related experiences and socioeconomic aspects compared with validated scales measuring symptoms of depression and anxiety, such as the HSCL-10. This kind of measure of mental health, which may be more sensitive to sociocultural variations, may be more appropriate to evaluate psychological distress during the present situation, as the appraisal of past mental health may be more influenced by personal factors such as memory bias and one's subjective perceptions.

### Limitations and future directions

There are several limitations to this study. First, the cross-sectional design prevents us from drawing any conclusions about causality. Longitudinal studies are needed to shed light on the trajectories of the sociocultural correlates of mental health during the COVID-19 pandemic. Second, our study used a convenience sample with a relatively low response rate (37%), and included a majority of participants with some college or a university degree; therefore, findings cannot be generalised to the larger Quebec population or to less educated populations. Third, differences may exist within the broad ethno-cultural groups used in the present study. Studies including larger samples and collecting more detailed ethno-cultural information are warranted. Fourth, we relied on self-reported items, and thus social desirability and response biases need to be taken into account. In particular, we used a measure of exposure to the virus that did not exclusively measure direct exposure to COVID-19, but rather whether the participant had tested positive or knew someone who tested positive for COVID-19. Future studies should investigate whether different types of exposure are differentially linked to mental health. Finally, our results cannot be generalised to different countries or to other Canadian provinces, and more research on regional and trans-national differences is needed.

In conclusion, despite its limitations, our study provides the first empirical evidence of the impact of sociocultural inequalities on mental health during the COVID-19 pandemic in the Canadian context. Public health authorities should acknowledge that pre-existing social and ethno-racial inequalities are exacerbated by the present pandemic, and actively monitor the evolution of the COVID-19 across sociocultural groups. Policies and messaging should be aimed at promoting inclusiveness at the societal level, to reduce the discrimination of racialised minorities, protect vulnerable groups and be better prepared for the second wave. The implementation and evaluation of multi-sectoral, community-based anti-discrimination programmes is warranted. Efforts should ensure that mental health services are accessible and culturally sensitive to racial minorities during, and in the aftermath of, the pandemic.

## Data Availability

The data-sets generated and/or analysed during the current study are available from the corresponding author, upon request.

## Supplementary material

For supplementary material accompanying this paper visit https://doi.org/10.1192/bjo.2020.146. click here to view supplementary material
