# Supplementary material for: Ethno-cultural disparities in mental health during the COVID-19 pandemic: a cross-sectional study on the impact of exposure to the virus and COVID-19-related discrimination and stigma on mental health across ethno-cultural groups in Quebec (Canada)
Source: BJPsych Open. 2020 Dec 9;7(1):e14. doi: 10.1192/bjo.2020.146 (PMC7844156; doi:10.1192/bjo.2020.146)
Supplement: Supplementary file 1 [file S2056472420001465sup001.docx]

**Supplemental material**

Table 1. Pearson correlation matrix of study variables (N=3,273).

| **Variables** | 1 | 2 | 3 | 4 | 5 | 6 | 7 | 8 | 9 | 10 | 11 | 12 | 13 | 14 | 15 | 16 | 17 |
| --- | --- | --- | --- | --- | --- | --- | --- | --- | --- | --- | --- | --- | --- | --- | --- | --- | --- |
| 1 Age | 1.00 |  |  |  |  |  |  |  |  |  |  |  |  |  |  |  |  |
| 2 Gender | -0.16 *** | 1.00 |  |  |  |  |  |  |  |  |  |  |  |  |  |  |  |
| 3 Ethnicity | -0.33 *** | 0.03 | 1.00 |  |  |  |  |  |  |  |  |  |  |  |  |  |  |
| 4 Religion | -0.12 *** | 0.02 | -0.05 ** | 1.00 |  |  |  |  |  |  |  |  |  |  |  |  |  |
| 5Language | -0.14 *** | 0.01 | 0.19 *** | 0.08 *** | 1.00 |  |  |  |  |  |  |  |  |  |  |  |  |
| 6 Generation | 0.24 *** | -0.04 * | -0.64 *** | 0.00 | -0.19 *** | 1.00 |  |  |  |  |  |  |  |  |  |  |  |
| 7 Education | -0.07 *** | -0.04 * | 0.14 *** | 0.07 *** | 0.09 *** | -0.20 *** | 1.00 |  |  |  |  |  |  |  |  |  |  |
| 8 Household size | -0.29 *** | 0.04 * | 0.25 *** | -0.05 ** | 0.05 ** | -0.21 *** | 0.01 | 1.00 |  |  |  |  |  |  |  |  |  |
| 9 Employment | -0.25 *** | -0.03 * | 0.07 *** | 0.05 ** | 0.06 ** | -0.07 *** | 0.19 *** | 0.07 *** | 1.00 |  |  |  |  |  |  |  |  |
| 10 Income | 0.05 * | -0.07 *** | -0.07 *** | 0.00 | 0.07 *** | 0.08 *** | 0.27 *** | 0.22 *** | 0.28 *** | 1.00 |  |  |  |  |  |  |  |
| 11 Geographical location | -0.08 *** | 0.03 | 0.27 *** | 0.10 *** | 0.24 *** | -0.28 *** | 0.13 *** | 0.08 *** | 0.00 | 0.01 | 1.00 |  |  |  |  |  |  |
| 12 Non-COVID related discrimination | -0.16 *** | 0.01 | 0.19 *** | -0.01 | 0.09 *** | -0.14 *** | -0.02 | 0.05 ** | -0.06 *** | -0.12 *** | 0.08 *** | 1.00 |  |  |  |  |  |
| 13 Mental health prior to pandemic | -0.21 *** | 0.11 *** | 0.04 * | 0.10 *** | 0.08 *** | 0.00 | -0.07 *** | 0.02 | 0.01 | -0.12 *** | 0.04 * | 0.15 *** | 1.00 |  |  |  |  |
| 14 Mental health impact | -0.24 *** | 0.16 *** | 0.10 *** | 0.05 ** | 0.07 *** | -0.04 * | -0.02 | 0.04 * | 0.04 * | -0.07 *** | 0.07 *** | 0.16 *** | 0.35 *** | 1.00 |  |  |  |
| 15 HSCL factor score | -0.25 *** | 0.16 *** | 0.14 *** | 0.04 * | 0.06 *** | -0.07 *** | -0.03 | 0.08 *** | 0.00 | -0.16 *** | 0.07 *** | 0.26 *** | 0.46 *** | 0.58 *** | 1.00 |  |  |
| 16 COVID-related discrimination | -0.17 | 0.00 | 0.13 *** | 0.02 | 0.08 *** | -0.11 *** | -0.04 * | 0.06 ** | -0.02 | -0.12 *** | 0.05 ** | 0.53 *** | 0.13 *** | 0.12 *** | 0.22 *** | 1.00 |  |
| 17 COVID-related stigma | -0.24 *** | -0.01 | 0.18 *** | 0.00 | 0.04 * | -0.14 *** | -0.02 | 0.08 *** | 0.04 * | -0.08 *** | 0.05 ** | 0.17 *** | 0.10 *** | 0.10 *** | 0.17 *** | 0.21 *** | 1.00 |

*p < .05; ** p < .01; ***p < .001
